# Pregnancy experiences of expectant parents with Neurofibromatosis type 1: a qualitative interview study

**DOI:** 10.1038/s41431-026-02099-6

**Published:** 2026-04-22

**Authors:** Gamze Kaplan, Debbie M. Smith, Ming Wai Wan, Hannah Slevin, Emma Burkitt-Wright, Shruti Garg

**Affiliations:** 1 Division of Psychology & Mental Health, School of Health Sciences, The University of Manchester, Manchester, UK; 2 Division of Cancer Sciences, School of Health Sciences, The University of Manchester, Manchester, UK; 3 Manchester Centre for Genomic Medicine, Manchester University NHS Foundation Trust, Manchester, UK; 4 Division of Evolution and Genomic Sciences, School of Biological Sciences, The University of Manchester, Manchester, UK; 5 Royal Manchester Children’s Hospital, Manchester University NHS Foundation Trust Manchester, Manchester, UK

**Keywords:** Quality of life, Human behaviour, Genetic counselling

## Abstract

Pregnancy in the context of Neurofibromatosis 1 (NF1) may be emotionally complex due to uncertainties surrounding inheritance and the condition’s variable presentation. This study aimed to explore how expectant parents with NF1 experience pregnancy and relate to their unborn child. Fourteen participants took part in individual semi-structured interviews, and data were analysed using reflexive thematic analysis. Participants described how decisions around conception and genetic testing were influenced by personal and medical history, perceived severity of NF1, and concerns about potential impact on their child. Participants described how ongoing uncertainty contributed to feelings of anxiety, guilt, and emotional restraint, which they managed through internal strategies such as seeking reassurance, information, and acceptance, as well as through external support networks. Notably, internal representations of the unborn child were sometimes shaped by cautious and emotionally regulated engagement in response to uncertainty. In a condition like NF1, where uncertainty may complicate prenatal bonding, adapting psychological interventions that have been used antenatally to promote early bonding and support later parenting outcomes could help strengthen emotional wellbeing and the developing parent-infant relationship.

## Introduction

Neurofibromatosis 1 (NF1) is a genetic condition affecting approximately 1 in 3000 individuals [1]. It follows an autosomal dominant inheritance pattern, meaning a child has a 50% chance of inheriting the condition if one parent is affected [2]. NF1 has a wide range of clinical features, including multiple café-au-lait macules, freckling in specific body regions, benign nerve tumours known as neurofibromas [3], and cognitive and behavioural issues, with an increased likelihood of neurodevelopmental conditions, such as ADHD and autism [4]. When inherited, its progression is unpredictable even within the same family [5] reflecting the absence of a clear genotype-phenotype correlation [6]. Pregnancy in NF1 can also present additional maternal risks, including neurofibroma growth, hypertensive disorders, vascular complications, and respiratory or cardiac concerns [7]. While various reproductive options available, including adoption, surrogacy, or remaining child-free, this article focuses on pregnancy regardless of conception method.

Studies on reproductive decision-making reported that expectant parents with NF1 consider not only the statistical likelihood of inheritance but also the subjective meaning of the condition shaped by their own experiences [8, 9]. These experiences influence how parents imagine their child’s future, including potential challenges in schooling, relationships, and everyday life [6, 10]. NF1 is therefore understood in relational and practical terms rather than probability alone [6, 11]. Within this uncertainty, parents engage in what has been described as “genetic responsibility”, making morally guided decisions for the unborn child while recognising that outcomes cannot be fully predicted [12].

Access to healthcare and information are also likely to shape reproductive and pregnancy experiences. In the UK, access to government funded reproductive treatment options, such as preimplantation genetic testing (PGT) is governed by strict eligibility criteria. While genetic counselling and service responsiveness can support decision-making [10, 13], pregnancy-related care is often marked by broader issues, including limited provider knowledge and restricted access to specialist support [10].

Previous NF1 research gave little attention to how individuals experience pregnancy itself once reproductive decisions are made. Pregnancy may heighten emotional strain due to concerns about maternal health, tumour progression, and unpredictable inheritance and presentation, fostering ongoing uncertainty and reduced perceived control [6, 7, 10, 14–17]. Such uncertainty may undermine parental wellbeing [18, 19] shape how parents relate to and imagine their unborn child [20, 21]. Pregnancy is also a period in which parents begin forming bonds with the unborn child [22, 23] with prenatal bonding associated with later parenting behaviours [24, 25] and child outcomes [26, 27]. However, in pregnancies involving a prenatal diagnosis of congenital diseases, maternal bonding may be compromised [28, 29].

To date, no research has explored how expectant parents with NF1 experience pregnancy or how they relate to their unborn child. Understanding these dynamics, may help services provide more responsive support during this period of heightened uncertainty, particularly within genetic counselling and antenatal settings. This study aims to explore how expectant parents with NF1 in the UK experience emotional uncertainty during pregnancy, and how they relate to their unborn child in this context.

## Method

### Design

A qualitative approach allowing exploratory, comprehensive, and flexible enquiry was taken [30]. Grounded in a critical realist ontology [31], we acknowledge the importance of understanding how expectant parents with NF1 construct meaning around pregnancy and bonding experiences, recognising both the shared and subjective dimensions of their realities. We adopted a neurodiversity-affirmed perspective, recognising that NF1 experiences are shaped by biological realities and broader structural contexts. NHS ethical approval was granted (Northwest Greater Manchester West 21/NW/0346) and the study was conducted between 01 March 2023 and 13 September 2024.

### Procedure

Participants were initially recruited through NHS clinics and NF charities via clinician or charity referrals, in the second stage, social media advertisements were included. Eligibility criteria included: (a) having a confirmed diagnosis of NF1 and is currently pregnant, being currently pregnant with a partner who has a confirmed diagnosis of NF1 or having a confirmed diagnosis of NF1 and being the partner of a person who is currently pregnant, and (b) speaking English to ensure verbal communication with the researcher. To capture a broad range of pregnancy experiences in the context of NF1 and given that this is the first study of its kind, no participants were excluded based on reproductive decisions, including use of prenatal testing or assisted reproductive technologies. Those interested in participating were contacted via email or phone to discuss the study and arrange a suitable time for the interview. An informed consent was obtained before the interview took place.

Semi-structured interviews were conducted individually with expectant parents. In three couples, both partners participated in the study and were interviewed separately due to the sensitive nature of the topics discussed. Participants were informed that interviews were confidential and focused on their own perspectives. A topic guide informed by prior literature and research team’s expertise, it covered: (a) pregnancy experiences, (b) bonding with their child, and (c) their role as a mother/father. The first four interviews were jointly conducted with HS, a clinician experienced in patient interactions, to support GK’s development. GK had prior qualitative interviewing experience and conducted the remaining interviews with ongoing supervision from DMS, a senior qualitative researcher. All but one interview was conducted online via Zoom, with one was conducted by telephone due to lack of internet access. Participants were reminded of their right to withdraw at any stage, and a distress policy was followed to ensure ethical sensitivity. Interviews were audio recorded, securely stored, and deleted after anonymised transcription.

### Data analysis and interpretation

**Fig. 1:**
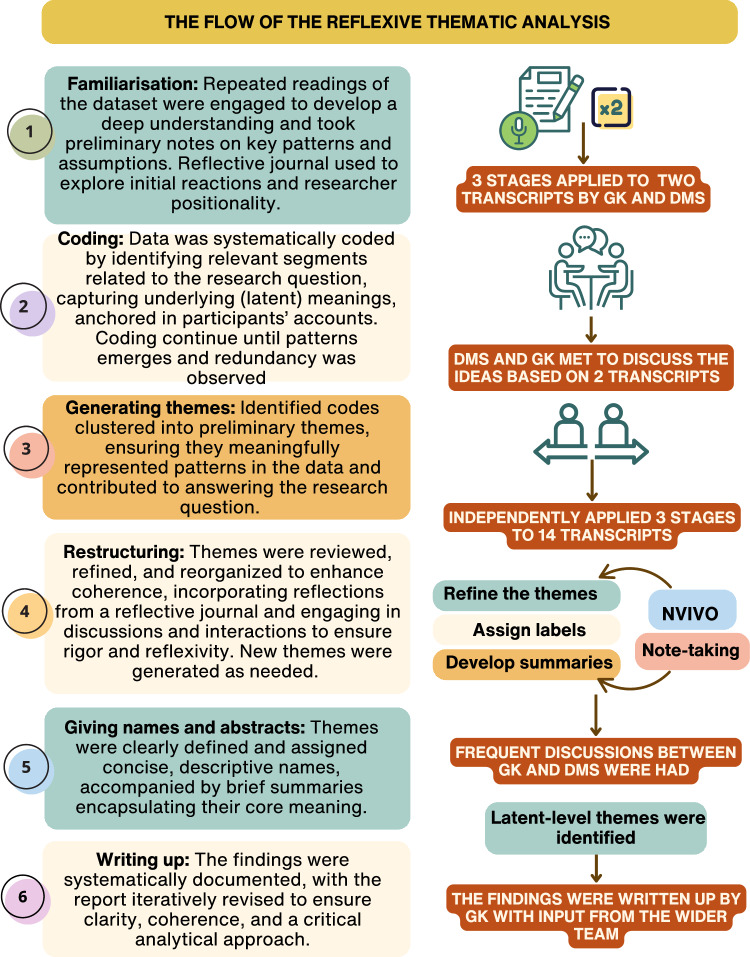
Detailed steps taken in reflexive thematic analysis [based on Braun & Clarke [24]]. The figure explains the six stages of reflexive thematic analysis: familiarisation, coding, generating themes, restructuring, defining and naming themes, and writing up. The right-hand side details how these stages were applied in the present study.

Transcribed data were analysed using a reflexive thematic analysis approach [32], through NVivo software (version 12; see Fig. 1 for more detail). Reflexive thematic analysis emphasises participants’ experiential insights and researcher reflexivity in theme creation [32]. An inductive approach was employed to explore a novel issue, and a latent level of analysis was applied to interpret underlying meanings and patterns, capturing broader influences.

The lead-researcher kept a reflective journal throughout, noting key insights, assumptions, decisions and personal development [32]. During analysis, reflective journal supported tracking of evolving interpretations and support the refinement of themes through critical engagement with data. The inclusion of multiple participants from the same family was considered during analysis to avoid over-representing experiences from a single family.

## Results

Fourteen expectant parents were interviewed between 8 and 37 weeks’ gestation. Interviews lasted a mean of 39.1 min (SD = 14.6; range = 21–68 min). Participants demographics are presented in Table 1. To ensure clarity and reflect participants accurately, we use the terms *mother/father with NF1* and *mother/father, partner* for expectant parents throughout the results. Additionally, we use the terms *‘baby’* and *‘child’* depending on the age being referenced, aligning with participant’s own terminology.

**Table 1:** Reported characteristics of interviewed expectant parents (*n* = 14).

Expectant parent	with NF1 *n* = 10 (8 female and 2 male) partner *n* = 4 (3 female and 1 male)
Conception choices	Conceived naturally *n* = 10 (waiting for chorionic villus sampling (CVS) testing *n* = 1) IVF with PGT *n* = 4
Term in pregnancy	First trimester *n* = 3 Second trimester *n* = 4 Third trimester *n* = 7
Parity	First time parent *n* = 10 Second time parent *n* = 2 Third time parent *n* = 2 (1 couple)
Age of NF1 diagnosis in the parent	During infancy (age around 1-2) *n* = 4 During childhood (age around 7-8) *n* = 6 During adulthood *n* = 1
Origin of the parental condition	Inherited from their family *n* = 3 Spontaneous mutation *n* = 7
Reported features of their pregnancy	Rapid growth in skin neurofibromas *n* = 2 Fatigue and sleep *n* = 1 Emotional fluctuations *n* = 1 Pain in backs and legs *n* = 1 No symptom *n* = 4

**Fig. 2:**
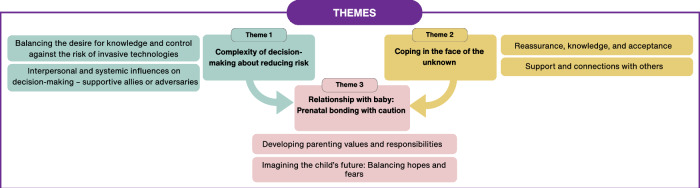
Three themes and sub-themes were created following the steps of reflexive thematic analysis. The figure presents the three main themes and their associated subthemes identified through the analytic process. Boxes represent themes and subthemes, and arrows illustrate the relationships between them.

Three themes and accompanying subthemes are presented below, supported by participant quotes and illustrated in Fig. 2: (a) *Complexity of decision-making about reducing risk*, (b) *Coping in the face of the unknown*, and (c) *Relationship with baby: Prenatal bonding with caution*.

### Theme 1. Complexity of decision-making about reducing risk

Most parents were aware of the 50% chance of their child inheriting NF1, with some also acknowledging the increased likelihood of neurodevelopmental conditions. This understanding was shaped by early information from family members and NF specialists over the years. When considering parenthood, most reported receiving additional information from their genetic counsellors and NF specialists. Views on the severity of the condition varied; while some parents thought how severely the child could be affected is uncertain, others who had inherited NF1 felt more at ease and drew on their own and their family’s experiences to perceive the condition as manageable. This complexity in decision-making is further explored in two subthemes:

#### Sub-theme 1. Balancing the desire for knowledge and control against the risk of invasive technologies

Four participants, including one couple and two solo parents, chose IVF in their pregnancy^1^. The decision was often framed as a way to take control and had done everything within their power to prevent their child from facing similar challenges.

*“we’ve at least done that one step, we’ve taken that one thing that we had that control over, out of the equation*.” (P07 – mother, partner).

IVF was not experienced as a straightforward path. Participants reflected on the physical intensity of the treatment and the stress of not knowing whether the early pregnancy would progress safely. One participant reflected on this early stage, describing how *“the pregnancy test says yes, but you just don’t know what’s actually going on inside your body… I was really struggling mentally, physically, because I was obviously still on medication as well for the IVF”* (P07 – mother, partner). Although early scans provided some reassurance, uncertainty persisted until discharge from the IVF clinic.

Prenatal testing, such as CVS, was a key consideration, though decisions were often influenced by the perceived risks of invasive procedures. Most parents stated that they chose not to test stating that termination was not an option, citing miscarriage risk and the emotional strain of knowing their child’s diagnosis during pregnancy. Others believed their child would likely be mildly affected, similar to themselves.

*“If that’s the case and the child would be like myself and like my mom or aunties and uncles or cousins, we really weren’t that worried if our child had NF… So we’ve decided to cancel that CVS testing”* (P05 – mother with NF1).

Only one expectant mother explained that they consider CVS:

“*They could be completely fine with the condition like me… But they could be completely different, and it could affect them quite severely… we’ll see if they had NF1, wouldn’t want to continue with the pregnancy”* (P11 – mother with NF1).

#### Sub-theme 2. Interpersonal and systemic influences on decision-making – supportive allies or adversaries

For some expectant parents, the decision-making to have a baby was described as a private process, while others reported as a shared decision involving wider families, one mother exemplified a more decisive influence recalling her mother’s initial advice to stay childfree and later prompting them reconsideration:

“*My mum has always been the reason… she just said just be happy with your husband and your cat*” (P12 – mother with NF1).

Discussions with clinicians about inheritance patterns and variability of NF1 helped participants understand their options and gave them space to think. Reassurance from genetics professionals helped them feel more confident about their decisions. Most parents did not consult their GP about NF-related issues, noting *“because he doesn’t obviously know, he’s not a specialist in it… And I don’t expect him to be”* (P06 – mother with NF1).

Several participants reported systemic challenges, such as delays and long waiting times. Structural barriers, such as NHS eligibility criteria and age limits shaped their decisions.

“*It was too soon to have a child with somebody… It’s by that point what you don’t want is we’re ready for a child, then apply and funding might not be available, it’s gonna take two years… so we just said, we’ll crack on with [PGT] now*” (P01 – mother with NF1).

### Theme 2. Coping in the face of the unknown

Uncertainty during pregnancy was described as an enduring emotional context rather than a transient response. Parents described holding multiple emotions simultaneously, feeling “*happy, excited, but nervous and a bit anxious*” (P08 – mother, partner with NF1). Distress centred on pregnancy progression, personal health, and the unpredictable inheritance and severity of NF1, driven by uncertainty about what the condition might mean for the child: “*It could be severely disfigured, it could be barely noticeable… it’s such a wide scope*” (P03 – father with NF1). Uncertainty was also closely tied to bodily change and future caregiving capacity, particularly for women with NF1: “*What if I get more tumours and it’s hard for me to physically look after them?”* (P10 – mother with NF1). These concerns extended to the unpredictable severity and lived impact of NF1 for the child, including appearance and long-term functioning, with feelings of guilt about the possibility of their child facing similar challenges. How parents navigated this emotional complexity is explored further across two sub-themes.

#### Sub-theme 1: Reassurance, knowledge, and acceptance

Participants described coping with uncertainty as an ongoing and effortful process, in which reassurance, knowledge, and acceptance were repeatedly mobilised. While some reassurance-seeking strategies resembled those commonly reported in pregnancy more broadly, in the context of NF1 they were experienced as fragile and short-lived, requiring sustained vigilance. Parents described closely monitoring bodily signs and clinical markers, such as foetal movement and appointments, while remaining uncertain about how to interpret them. Reassurance associated with clinical care was similarly transient:

“*An appointment’s quite reassuring and then the next time an appointment is coming up it’s like… more of an anxious time*” (P08 – mother, partner).

For participants with NF1, reassurance-seeking extended beyond foetal *wellbeing* to heightened attention to their own bodies. Several mothers described ongoing aches and pains, particularly in the back, hips, and legs, often linked to existing NF1-related conditions, such as scoliosis or nerve involvement, described a sustained watchfulness toward bodily change. One mother described rapid tumour increase across pregnancies:

“*Before I got pregnant, I had two tumours… In this pregnancy I’ve got nineteen at the moment*” (P04 – mother with NF1).

Knowledge was used to bring structure to uncertainty in different ways. For some, particularly those with prior experience of NF1, knowledge was grounded in familiarity and preparedness.

“*The more experience you’ve got so you know what to look out for… is there cafe-au-lait marks and visual problems et cetera… having it and having a child that’s gone through it gets you better prepared*” (P03 – father with NF1).

For others, knowledge was sought through healthcare professionals as a form of psychological preparation.

“*I would rather have the statistical information… to say, look, obviously we hope that everything is going to be absolutely fine… but there’s a 25% chance that this could happen*” (P08 – mother, partner).

Acceptance was described as a deliberate strategy for containing worry rather than resolving uncertainty. Participants emphasised recognising the limits of control during pregnancy, while remaining emotionally engaged with the process.

A few participants drew on their own or family experiences of NF1 to narrow uncertainty about their child’s future, treating personal experience as a reliable guide to severity or inheritance. For a few others experience-based reasoning was used to anticipate inheritance itself, based on family patterns or previous children.

“*But I do expect my child to be born with the NF gene, considering my first born with it as well*” (P13 – mother with NF1).

While such accounts sometimes reflected misconceptions about NF1 inheritance and variability, they appeared to function as emotionally protective strategies, reducing the range of imagined outcomes during pregnancy.

#### Sub-theme 2: Support and connections with others

Social connections were important for coping with uncertainty. These included partners, family, healthcare professionals and, for some, social media. These connections helped contain emotional demands and sustain engagement with pregnancy. Partners were described as the primary source of support for most participants. For men with NF1, partners played a key role in helping them manage feelings of guilt and responsibility associated with the possibility of passing on the condition, often through open discussion and a shared sense of facing uncertainty together.

“*Just open discussions… about worst-case scenario and how we’ll deal with it… we just deal with it as a family*” (P03 – father with NF1).

For women with NF1, it was often related to partners’ practical involvement, emotional containment, and efforts to minimise stress during pregnancy, sometimes by withholding their own worries.

“*He doesn’t really show [his worry] towards me, because he doesn’t want me to get any stress*” (P13 – mother with NF1).

For some, family provided essential support, especially for those who grew up in nurturing environments and reflected a hope to offer their child a similar sense of security and independence. Others described being physically and emotionally distanced from their families, due to experiences like domestic abuse. In these instances, they had an increased reliance on external support.

Healthcare professionals were frequently described as supportive when participants felt understood and taken seriously, particularly genetics teams and NF specialist.

“*My NF nurse… she understands more of my concern than my GP does… if there’s been any issues, she’s been really supportive*” (P06 – mother with NF1).

In contrast, some described limited professional knowledge, administrative difficulties, and poor communication and coordination as adding to emotional demands and requiring ongoing self-advocacy.

Social media platforms offered mixed experiences. For some, platforms like Instagram and TikTok provided connection, validation, and a sense of community. However, some found online groups overwhelming, noting that the focus on severe or complex cases often heightened their anxiety about the potential severity of NF1.

*“I have to come away from them sometimes because, the good, there are great sorts of help but at the same time, it tends to be the very complex patients and parents”* (P02 – mother, partner).

### Theme 3: Relationship with baby: Prenatal bonding with caution

Expectant parents described diversity in how they related to their unborn baby. For some, bonding developed through emotionally open, embodied interactions, such as feeling movements, talking, noticing responsiveness, and imagining shared futures: “*We sort of like connecting her through that… I just talk to her… and she just sort of reacts to my voice*” (P06 – mother with NF1). For others, bonding was present but deliberately moderated in response to uncertainty. This involved active emotional regulation rather than detachment. For some, uncertainty persisted despite testing or treatment, as illustrated by one mother who described genetic testing as “*only 99% accurate… there’s still a chance*” (P10 – mother with NF1), highlighting how tenderness and caution co-existed. While practices, such as delaying purchases or keeping the pregnancy private are common in pregnancy more broadly, they reflected heightened efforts to balance hope and caution under NF1-related uncertainty. These bonding is explored further across the following sub-themes:

#### Sub-theme 1. Developing parenting values and responsibilities

Expectant parents with NF1 expressed a desire to build a close connection, where their child felt comfortable sharing without fear, yet within defined boundaries that provide safety and structure. Many drew inspiration from their own parents, aspiring to replicate positive aspects of their upbringing while rejecting overly controlling or restrictive practices they experienced. Openness was viewed as central, particularly when discussing about NF1.

“*Why are we a bit different, why do we have to go to hospital… I would just rather have a very open and honest explanation of what these are, what might happen, so they got an understanding.”* (P02 – mother with NF1)

Planned parenting arrangements reflected not only divisions of care but also pre-planning around physical capacity. One mother with NF1, who used crutches, described carefully adapting home environment to reduce risk:

“*If I’m going up and downstairs, baby will go in the carrier… I can hold the banister, and if I fall, the baby’s on me and protected*” (P01 – mother with NF1).

#### Sub-theme 2. Imagining the child’s future: balancing hopes and fears

All parents emphasised the importance of their children growing up happy and healthy, alongside concerns about NF1-related uncertainty. These concerns centred on unpredictable range of possible outcomes, and related medical challenges, including the burden of ongoing appointments and potential complications. Many expressed hope that their child would not inherit NF1 or, if they did, would be only mildly affected. For some, this meant a life free from severe health issues, to enjoy everyday experiences like working, driving, and forming relationships: “*just a healthy life, with no other complications*” (P12 – mother with NF1).

Expectant parents with NF1 envisioned their child in different ways. Some focused on physical features, such as “*really long beautiful hair*” (P04 – mother, NF1*)* or a “*chunky” (P09* – father with NF1*)* appearance, while others imagined shared experiences like “*going on walks and playing on the field*” (P14 – father, partner). However, not all had formed clear images of their children.

Many parents worried about societal pressures, particularly bullying or exclusion. Some recalled personal experience of being bullied at school due to their physical features, such as being *“told to wash my neck [due to the freckles]*” (P12 – mother with NF1) and feared that their child might face similar hardships. Despite these concerns, parents emphasised happiness, autonomy, and meaningful relationships over achievement: “*I don’t really care if she doesn’t get a job… as long as she is happy and healthy*” (P04 – mother with NF1).

## Discussion

**Fig. 3:**
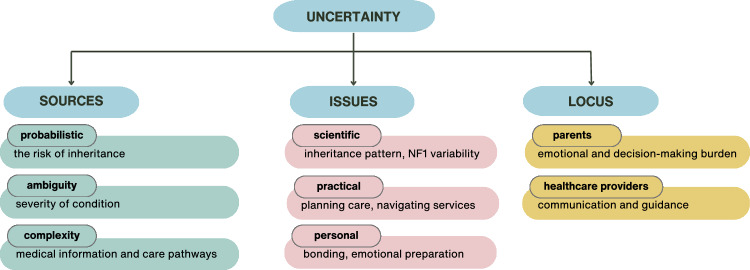
Han et al.’s [25] taxonomy of uncertainty applied to the experiences of expectant parents with NF1. The figure shows the multidimensional nature of uncertainty, categorising uncertainties by distinguishing between scientific, practical, and personal forms arising from different sources, issues, and residing in different loci.

This study explored lived experiences of expectant parents with NF1, and findings demonstrate that rather than identifying a single dominant response, uncertainty was managed through combinations of decision-making strategies, ongoing appraisal and regulation of uncertainty through reassurance-seeking, acceptance, and vigilance during pregnancy, and carefully calibrated and revisited approaches to prenatal bonding. Uncertainty was experienced as an embodied, relational, and systemic one, shaped by pregnancy-related risks, healthcare encounters, and family dynamics. These findings can be mapped onto Han et al.’s [33] integrative taxonomy of uncertainty in healthcare, which illustrates the sources, issues, and locus of uncertainty relevant to expectant parents with NF1 (see Fig. 3) to visualise how different types of uncertainty shaped participants’ emotional responses and decision-making.

During pregnancy, parents’ internal representations of their unborn child were shaped through ongoing processes of prenatal bonding characterised by emotional complexity, including the co-existence of joy, anticipation, concern, and vigilance. Parents described engaging in widely reported pregnancy practices—such as monitoring bodily signs, valuing scans, delaying preparations, or keeping the pregnancy private until later milestones [34–39] —which, in the context of NF1, were further influenced by prolonged uncertainty around genetic variability and future implications. Similar to other inherited genetic conditions, such as retinoblastoma, their reproductive decisions were shaped by fears of transmission, uncertainty about severity, and the emotional implications of testing and pregnancy management [13, 40].

While research in other genetic conditions suggests that familiarity with disability can heighten perceived risk [41], for several expectant parents with NF1 in this study, experiential familiarity instead functioned as a normalising influence, narrowing imagined futures. Similar patterns have been noted in NF1 research, where familiarity may also lead individuals to downplay potential severity [8, 42]. Here, such interpretations appeared less about denial and more about stabilising expectations and maintaining emotional engagement during pregnancy [43]. These behaviours can be interpreted through Uncertainty Management Theory [44], which suggests that individuals do not always aim to reduce uncertainty; rather, they may tolerate or manage it to preserve emotional stability. In this context, moderated emotional engagement may have served as an adaptive response rather than emotional disengagement.

Partners were the primary source of support for most participants. The sense of togetherness helped them manage the emotional and practical challenges of pregnancy. This aligns with Hickerton et al. [45], highlighting partner involvement as a source of resilience and relationship strengthening in families continuing a pregnancy with a known or likely genetic condition. However, support systems varied. Some drew strength from close ties, while others, including solo parents or those distanced from their families, relied on external sources of support. These differences shaped coping approaches, highlighting the need to recognise and respond to the specific needs of those without close familial or partner support, who may benefit from more individualised and proactive care during pregnancy.

Engagement with wider communities, particularly online, was selective and mixed. While some parents found reassurance, validation, and a sense of connection, others reported increased anxiety when exposed to more complex NF1 presentations. This pattern reflects how shared biological conditions can shape belonging unevenly, with parents tending to relate more easily to experiences they perceived as similar to their own, while distancing themselves from others [46, 47]. As a result, parents often balanced engagement with avoidance, consistent with evidence that seeking information can sometimes increase uncertainty when content is overwhelming or conflicting [48–50].

### Strengths and limitations

Most participants perceived themselves as mildly affected, which may indicate a participation bias, as individuals who view their condition as more severe may be less inclined to participate during pregnancy. As most were first-time parents, the findings may reflect a particular set of emotional and informational needs that differ from those with previous pregnancies. Demographic characterisation of participants was limited to information obtained through recruitment communications and interviews, which may have restricted the contextual detail available for interpreting the findings. Diagnostic status was based on participants’ self-report of a clinical diagnosis of NF1, and molecular confirmation was not obtained; we therefore cannot exclude the possibility of related conditions with overlapping features (e.g., Legius syndrome).

A key strength of the study is its focus on a critical yet underexplored period, pregnancy in the context of NF1. This focus aligns with wider public health priorities, including the United Nations [51] Sustainable Development Goal 3, which promotes good health and well-being, and the UK Government’s [52] vision for the first 1001 days, which emphasises seamless, early support for families. The inclusion of varied conception pathways (e.g., natural conception and PGT) also enriched the findings and increased representativeness, since these options are publicly funded in the UK.

### Implications for practice

Participants described mixed experiences with healthcare professionals, who were valued sources of support but at times also contributed to stress when communication was inconsistent. Clear, accessible information and better coordination across services may therefore help families feel more supported. The role of NF specialists was consistently valued, suggesting that this UK-based model of care may offer a transferable example for other health systems.

Finally, in the context of NF1, where uncertainty about a child’s future can complicate emotional connection during pregnancy, structured interventions like Baby Triple P [53], which has been adapted for antenatal settings, could help parents build confidence, regulate anxiety, and strengthen early bonding.

### Implications for future research

Although this study focused on the pregnancy period and early bonding, it also offered insights into experiences with reproductive technologies. The psychological and social implications of options, such as PGT for NF1 can be explored further. Longitudinal qualitative studies can further explore how expectant parents’ experiences evolve during and after pregnancy, particularly in terms of their psychological adjustment and relationship with their child.

## Conclusion

To our knowledge, this is the first study to explore the lived experiences of expectant parents with NF1. The findings show how genetic uncertainty contributes to emotional complex pregnancies in which early bonding is present but often cautiously moderated, revealing limitations in standard care models and the need for more psychologically responsive support.

## Data Availability

The data used in this study can be available from the corresponding author on reasonable request.

## Footnotes

1 The “IVF path” refers to the use of IVF in combination with preimplantation genetic testing (PGT). This process involves creating embryos through IVF and genetically testing them for the condition before implantation. See: Genomics Education Programme – Preimplantation Genetic Testing
